# Cranio-Mandibular Disorders after Whiplash Injury: A Mono-Institutional Clinical Study on 31 Patients

**DOI:** 10.3390/ijerph19020901

**Published:** 2022-01-14

**Authors:** Massimo Corsalini, Saverio Capodiferro, Fabio dell’Olio, Giovanni Albanese, Nicola Quaranta, Biagio Solarino, Santo Catapano, Daniela Di Venere

**Affiliations:** 1 Department of Interdisciplinary Medicine, University of Bari “Aldo Moro”, p.za G. Cesare 11, 70124 Bari, Italy; f.dellolio.odo@outlook.it (F.d.); giannialbanese69@libero.it (G.A.); biagio.solarino@uniba.it (B.S.); daniela.divenere@uniba.it (D.D.V.); 2 Department of Basic Medical Sciences, Neurosciences and Sense Organs, University of Bari “Aldo Moro”, p.za G. Cesare 11, 70124 Bari, Italy; nicolaantonioadolfo.quaranta@uniba.it; 3 Dental School, Dental Clinic, University of Ferrara, c.so Giovecca, 203, 44121 Ferrara, Italy; santo.catapano@unife.it

**Keywords:** whiplash, temporo-mandibular disorders, accidents, neck pain, post-traumatic stress symptoms

## Abstract

Background: Whiplash is a consequence of traumatic injuries, mostly related to road accidents, with variable clinical manifestations, also known as Whiplash Associated Disorders, such as neck, head and temporo-cranio-mandibular pain. Methods: The current study aims to evaluate the onset and evolution of temporomandibular joint pain in people with whiplash in a study group treated with the use of Zimmer Collars (adjustable rigid cervical collars for neck immobilization), as compared to a control group. This prospective study included 31 patients followed by the Dental Prosthesis Department of the University of Bari “Aldo Moro”: 20 patients with whiplash (age range: 20–39 years) treated with Zimmer collars and 11 patients with whiplash (age range: 20–33 years) who were not. Immediately after the whiplash occurred, a visual analogue scale (VAS) was used to describe the intensity of pain and to complete the chart of the European Academy of Craniomandibular Disorders. Five out of twenty patients, already treated with a Zimmer collar, wore an occlusal splint as well because of persistent pain reported at the 28-day and 60-day follow-up and were supported by pharmacological therapy with analgesics (paracetamol) and muscle relaxants (thiocolchicoside). Results: During the last follow-up (at six months), three out of five patients displayed a residual VAS score of 3, 4, and 5, respectively, while the remaining two displayed a VAS of 0. In the control group, four out of eleven patients needed to wear an occlusal splint but without muscle relaxants and analgesics pharmacological therapy; these four corresponded to the patients showing a residual painful symptomatology, with VAS reaching value of 2, and also were the oldest patients of the group. Data regarding VAS values and Zimmer collar use, both at the first visit and six months later, were statistically analyzed. Conclusion: Our prospective study highlights how whiplash-associated acute disorders are often self-limiting over a period of few months, thus reducing the possibility of symptom chronicity; the latter seems to be strictly related to lesion severity, pre-existence of a craniomandibular dysfunction and patient age, but appears to be independent from Zimmer collar use, as statistically confirmed.

## 1. Introduction

The current definition of whiplash as a transfer mechanism of collision energy towards the neck due to a sudden acceleration/deceleration was introduced in 1995 by the Quebec Task Force on Whiplash Associated Disorders (WADs) [1]. Vehicle collision or different types of accidents, such as diving, are generally recognized as the mostly frequent causes, as the impact can damage soft and hard tissues of the concerned area with different clinical manifestations, as listed in Table 1 [1].

Whiplash-related clinical manifestations can occur within different timeframes post-trauma: Whiplash Syndrome appears immediately or in the following 72 h, while Late Whiplash Syndrome appears at least 6 months later. The main symptom is neck and head pain but also temporo-mandibular joint (TMJ) or cranio-mandibular pain [1].

Data emerging from the international literature on temporomandibular disorders in whiplash patients are quite contradictory. Salè et al. [2] reported an increased risk of developing chronic temporomandibular disorders (TMDs) one year after the traumatic event in one out of three whiplash patients reporting cervical pain. Marini et al. [3] examined 65 patients with orofacial pain previously diagnosed as late whiplash syndrome and compared them with a control group; they observed that patients displayed a higher myofascial pain frequency and disk displacement with reduction. In addition, Salé et al. [4] evaluated a group of 60 patients over a 15-year period after whiplash trauma by temporomandibular joint MRI and observed a greater prevalence of abnormalities compared to the control group. Conversely, whiplash is not considered a major risk factor for development of TMDs by several authors [5,6].

Pérez del Palomar et al. [7] used a dynamic 3D model to evaluate the frontal and lateral impact during whiplash-caused trauma and concluded that such stress of osteoarticular and ligamentous structures does not display an adequate intensity to justify WADs symptomatology and symptom chronicity. Hani Abdul et al. [8] described the arthroscopic findings in 30 patients who had suffered WADs, highlighting that nonspecific visual evidence for direct trauma and articular tissue damages caused by common chronic degenerative processes was detectable, thus concluding that pre-existing asymptomatic conditions could exacerbate and reveal whiplash symptomatology.

Notably, it has been reported in the literature that victims of motor accidents in Lithuania did not report chronic symptomatology even after acute whiplash injury [9]; on the contrary, victims of the same trauma in western countries frequently report chronic symptomatology, probably due to the socio-cultural and medico-legal factors leading to financial compensation [10].

The current study aims to evaluate pain in the TMJ as a symptom in whiplash patients, both immediately after the trauma and six months post-injury, and to compare symptoms in patients needing to wear the Zimmer collar and those who do not.

## 2. Materials and Methods

We studied 31 patients (22 females and 9 males) treated at the Dental Prosthesis Department of the University of Bari “Aldo Moro”. Precisely, 20 patients (age range 20–39 years) were treated using a Zimmer collar, and a control group of 11 patients (age range 20–33 years) did not receive a Zimmer collar, after whiplash. Patients received an occlusal splint (with an increased vertical dimension and facilitating anterior dislocation of the mandible) when persistent pain was reported at a 28 days follow-up, along with paracetamol and thiocolchicoside, when necessary. Patients were clinically examined immediately after whiplash trauma and VAS was used to describe the intensity of the referred pain [11]; then, the chart of the European Academy of Craniomandibular Disorders was completed, including patient history along with signs and symptoms of possible craniomandibular disorders according to the RCD classification [12]. The same expert clinician followed up with patients within a few hours after trauma, and subsequently seven days, four weeks, sixty days, and finally six months post-trauma. The mean age of patients who received a Zimmer collar was 29.75 ± 7.20 years, vs. 28.64 ± 4.27 for the control group. Statistical analysis was conducted using IBM SPSS Statistics for Windows, Version 27.0. Each statistical test used in this study (paired sample *t*-test, independent sample *t*-test, χ^2^ test) had a significance level of *p* < 0.05.

## 3. Results

**Figure 1:**
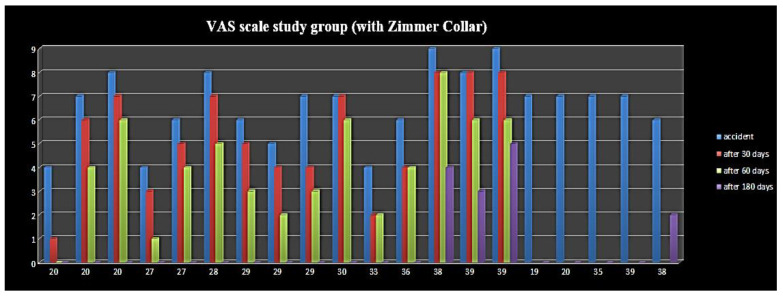
VAS scale study group (with Zimmer Collar).

**Figure 2:**
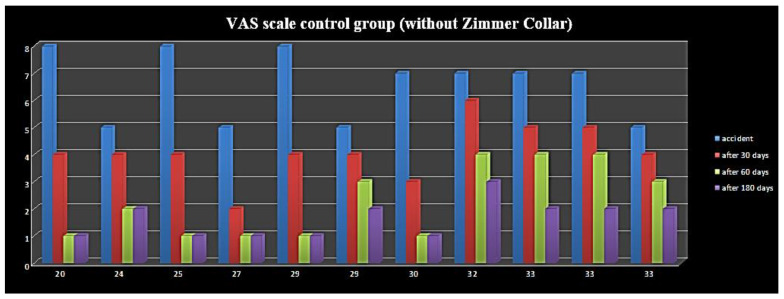
VAS scale control group (without Zimmer Collar).

**Figure 3:**
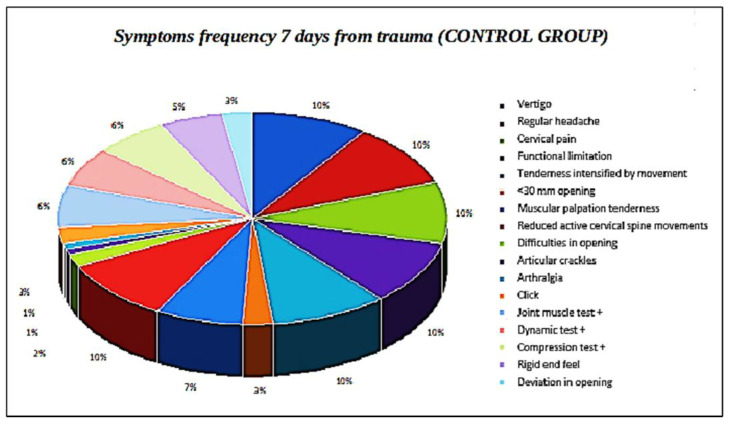
Symptom frequency 7 days from trauma (control group).

**Figure 4:**
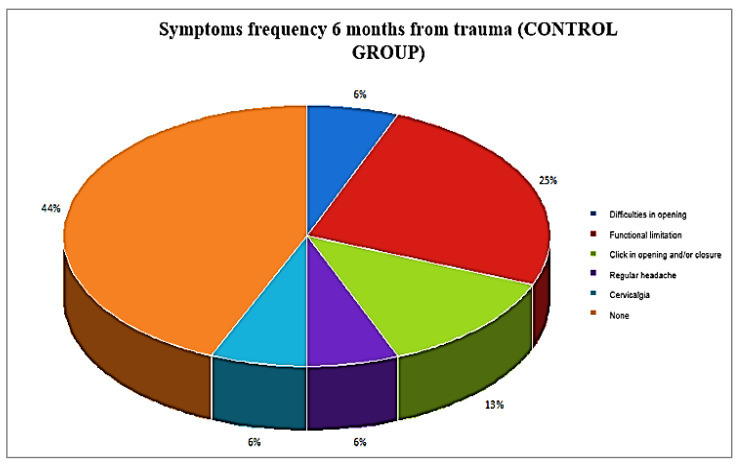
Symptom frequency 6 months from trauma (control group).

Five out of twenty patients, already treated with a Zimmer collar (Table 2), received an occlusal splint, as persistent pain was reported at the 28-day and 60-day follow-up; they were also supported by analgesics and muscle relaxants. At the last follow-up (at six months), three out of five patients displayed a residual VAS of 3, 4, and 5, respectively, while the remaining two patients displayed a VAS of 0 (Figure 1). Furthermore, patients with symptom persistence were older (38 and 39 years old); the RCD classification values at six months was almost absent, and only four patients displayed values of 1A. In the control group (Table 3), four out of eleven patients needed to wear an occlusal splint, but without muscle relaxants and analgesics pharmacological therapy; these patients showed residual pain, with VAS reaching a value of 2, and also were the oldest patients of the group (Figure 2 and Figure 3). The values according to RCD classification after six months were displayed by only three patients, with a value of 1A (Figure 4).

Fifteen out of twenty patients (75%) of the study group reported pain absence at the last follow-up.

**Figure 5:**
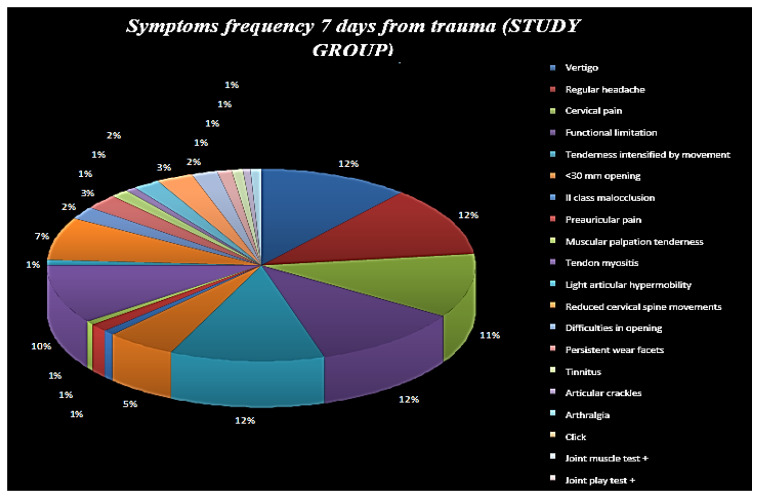
Symptom frequency 7 days from trauma (study group).

**Figure 6:**
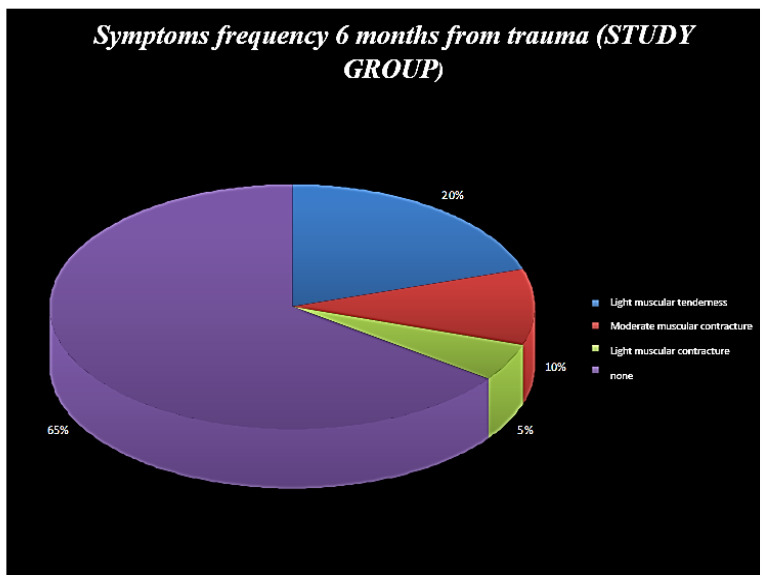
Symptom frequency 6 months from trauma (study group).

Twenty patients of the study group (100%) presented myositis-affecting tendons of the masticatory muscles due to contracture after whiplash trauma. By forcing the condyle in a superolateral or posterior superolateral position, the collar fosters anteromedial dislocation of the meniscus and retro-discal tissue compression, with consequent posterior retro-discitis and capsulitis (Figure 5 and Figure 6). Four out of five patients, still enduring painful symptomatology, displayed VAS values of 2, 3, 4, and 5, respectively, with the latter being unacceptable and requiring further evaluation. In addition, at the first follow-up, three out of twenty patients exhibited evidence of wear facets over the dental surface, suggesting the diagnosis of parafunctional activities such as clenching and grinding. Consequently, the previous subgroup (three patients) consisted of two partially healed patients.

With regard to the results of the statistical analysis, during the first visit, the mean VAS score was 6.45 ± 1.39 points; after six months, the mean score was significantly reduced to 0.70 ± 1.53 points (mean difference, m.d. = −5.75; *p* < 0.001). A similar result was observed in the control group (m.d. = −5.82; *p* < 0.001): mean VAS decreased from 6.55 ± 1.29 to 0.73 ± 1.01. Furthermore, the difference in mean six-month VAS score between groups was not significant (*p* = 0.958), thus meaning that Zimmer collar therapy did not improve the prognosis of patients and that whiplash acute disorders are mainly self-limiting over six months.

During the first visit, eight patients (40.00%) had a single RCD symptom and 12 (60.00%) had two RCD symptoms in the group receiving the Zimmer collar, against seven (63.64%) and four (36.36) in the control group, respectively; in both groups, nobody was asymptomatic. During the six-month follow-up visit, in both groups there were no patients with two RCD symptoms, and the number of asymptomatic patients grew to 15 (75.00%) in the group that received the Zimmer collar and to eight (72.73%) in the control group, but this difference was not significant (*p* = 0.890), thus confirming that Zimmer collar therapy did not improve the prognosis of whiplash acute disorders, which self-limited over six months.

To better explain the reasons of symptom chronicity, the authors observed that such conditions could be related to the severity of the initial lesions, the pre-existence of TMJ disorders, and age. Among all patients involved in the study, 15 (48.39%) had an RCD symptom and 16 (51.61%) had two RCD symptoms initially; after six months, only eight patients showed chronicity of symptoms.

The correlation between these outcomes was positive, moderate in strength, and statistically significant (Phi coefficient = 0.571, *p* = 0.001), confirming the authors’ observations. The same findings were observed for the correlation between symptom chronicity and the pre-existence of TMJ disorders (Phi coefficient = 0.555; *p* = 0.002). The correlation between age and chronicity of symptoms was positive, weak, and not significant (point-biserial correlation coefficient, r = 0.313; *p* = 0.086); three patients with a residual VAS score higher than 3 were older than 38 years.

## 4. Discussion

Whiplash-associated clinical manifestations may appear after a variable timeframe post-trauma and therefore are categorized as Whiplash Syndrome or Late Whiplash Syndrome. The clinical scenario is extremely variable, as different signs and symptoms can interact in different ways. Symptoms and disorders encountered include headache, pain and cervical rigidity, shoulder pain, back pain, craniomandibular disorders, para-aesthesia, vertigo, visual disorders, and dizziness.

In addition, acute and chronic manifestations can variably be developed. With regard to the acute symptomatology, patients are able to perceive little or no pain immediately after the injury; symptomatology gradually increases in the following days, probably due to oedema diffusion within the soft tissues [10,11,12,13]. Limitation of the cervical movements, cervical tension, muscular spasm, and/or tumefaction may accentuate a few hours after the trauma.

Whiplash-associated disorders are often self-limiting over time, and many patients undergo only observation in the first months. Lampa et al. [14] observed that symptoms of craniomandibular disorders developed within a week following the motor accident in the majority of patients, with a timeframe ranging from one month to five years after the accident [15]. Garcia et al. [16] used magnetic resonance imaging to examine TMJ abnormalities in 95% of 87 cervical whiplash patients who presented TMJ symptoms but reported no direct trauma to the face, head or mandible, and no TMJ complaints prior to the trauma.

Furthermore, some elements of whiplash treatment, such as a cervical collar (Zimmer collar) and/or physiotherapy tractions, may be potentially involved into the pathogenesis of craniomandibular disorders; the forcing of the condyle in a superolateral or posterior superolateral position allows for anteromedial dislocation of the meniscus with retro-discal tissue compression and consequent posterior retro-discitis and capsulitis.

Although still controversial, authors generally agree that whiplash can cause more severe symptoms (especially in the short term) in contrast to non-traumatized patients. Other authors [17,18,19,20,21] have reported that post-traumatized patients with TMJ disorders displayed increased neck pain frequency as well as greater head and face, masticatory muscle, and TMJ pain in comparison to non-traumatized patients [3]. Krogstad et al. [22] report that post-traumatized TMD patients were unresponsive to treatment and required additional methods in comparison to non-traumatized patients. De Boever et al. [23] compared two groups of TMD patients: one with history of head and neck trauma and the other with no history correlated to trauma. The trauma group initially showed more noticeable symptoms, but both groups responded well to conservative treatment, as shown one year later when re-evaluated with the Helkimo dysfunction index.

Generally, negative prognostic factors towards functional recovery and TMD onset are the following: high impact speed (>60 Km/h), elevated initial pain intensity, head position during impact, headrest absence, advanced age, female sex (genetic or behavioral factors could entail minor muscular and structural resistance of female gender associated with greater hormonal-dependent hypermobility and smaller stress phenomena adaptation that favor central sensitization and the possibility of chronic pain), cervical rachis deformity, and ongoing legal cases.

Davis [24] claims that trauma-caused whiplash TMDs evolve into chronic pain, sustained by a neuroplasticity mechanism over different neuronal structures, and are thus responsible for nociception amplification and exaggerated pain response, with a noticeable decrease of pain threshold. Chronic pain is unrelated to the current actual damage of the articular structure, since the transmitted energy is insufficient to provoke true permanent lesions [24,25,26].

The current study highlights that the acute phase immediately after whiplash usually improves and can resolve within a few months, with the reduced possibility of chronicization. The residual symptomatology can persist for various reasons, as follows:

(A) Lesion severity: such as manifestation of a traumatic brain injury or craniocaudal joint dislocation. Indeed, a VAS of 4 remained in the patient that reported a traumatic brain injury and a VAS of 3 and 5 remained in those patients subject to very strong trauma.
(B) Pre-existent craniomandibular dysfunction: Two patients out of three with a residual VAS higher than 3 showed signs of parafunction at the six-month follow-up period.
(C) Patient age: three out of five patients with a residual VAS higher than 3 were older than 38 years; therefore, such patients were referred for longer therapy and gum shield use.
(D) Control group: in the short term, Zimmer collar therapy promotes tendon myositis, with greater pain reported with respect to the control group (no Zimmer Collar); furthermore, chronic pain after six months was equal between the study and control group in terms of percentage, thus highlighting how pain persistence is due to a central sensitization phenomenon affecting the nervous system.

The pathophysiology of TMD development and its exacerbation as a sequela of whiplash injury can be distinguished from other TMDs (caused by functional habits or microtrauma, e.g., parafunctions or malocclusion), as individuals have both a variable intensity macro-trauma to muscles, ligaments, and tendons of the cervical area and an adjunctive involvement of the central nervous system because of diffuse axonal injury [27,28] or resulting from prolonged masticatory muscle pain. In addition, the frequent experience of emotional trauma from injury may further contribute to create a complex neuropathology of pain exacerbation in TMD patients after whiplash [29].

## 5. Conclusions

Data from the literature on TMD amplification or exacerbation in whiplash injuries are quite discordant, as several factors may influence or complicate such an association as well its therapy, duration, and prognosis. Despite its limitations, especially regarding the low number of cases analyzed within, the current study demonstrates that whiplash-associated acute disorders are often self-limiting over a period of few months; symptom chronicity appears mostly related to lesion severity, possible pre-existence of craniomandibular dysfunction/disorder, and patient age, but on the whole is not related to Zimmer collar use.

## Figures and Tables

**Table 1:** Clinical classification of whiplash-associated disorders.

Grade	Clinical Presentation *	Subgrade
0	No complaint, no physical signs	2A: point tenderness. normal range of movement
1	Neck pain, stiffness, or tenderness	
2	Neck complaint and musculoskeletal signs: decreased range of movement and point tenderness	
3	Neck complaint and neurologic signs: decreased or absent deep tendon reflexes, weakness, or sensory deficits	2B: point tenderness. abnormal range of movement
4	Neck complaint and fracture or dislocation	

* deafness, dizziness, tinnitus, headache, memory loss, dysphagia, and temporomandibular pain can appear in all grades.

**Table 2:** Study group results (Zimmer collar users).

PT No.	AGE	SEX	VAS I Visit	VAS 6 Months	RDC I Visit	RDC 6 Months	Michigan Occlusal Splint	Zimmer Collar
1	20	F	4	0	1B + 3A	1A	NO	YES
2	20	M	7	0	1B	0	NO	YES
3	20	F	8	0	1B + 3A	0	YES	YES
4	27	M	4	0	1B	0	NO	YES
5	27	F	6	0	1B + 3A	0	NO	YES
6	28	F	8	0	1B + 3A	0	NO	YES
7	29	F	6	0	1B	0	NO	YES
8	29	F	5	0	1B + 2A	0	NO	YES
9	29	F	7	0	1A	0	NO	YES
10	30	F	7	0	1B + 3A	1A	YES	YES
11	33	F	4	0	1B	0	NO	YES
12	36	F	6	0	1B + 3A	0	NO	YES
13	38	F	6	4	1B + 3A	1A	YES	YES
14	39	F	8	3	1B + SA	1A	YES	YES
15	39	F	9	5	1B + 3A	1A	YES	YES
16	19	F	7	0	1B	0	NO	YES
17	20	F	7	0	1B	0	NO	YES
18	35	M	7	0	1A + 1B	0	NO	YES
19	39	M	7	0	1A + 1B	0	NO	YES
20	38	F	6	2	1A	0	NO	YES

**Table 3:** Control group results (Zimmer collar non-users).

PT No.	AGE	SEX	VAS I Visit	VAS 6 Months	RDC I Visit	RDC 6 Months	Michigan Occlusal Splint	Zimmer Collar
1	20	F	8	0	1B	0	NO	NO
2	24	M	5	2	1B	0	YES	NO
3	25	M	8	0	1B	0	NO	NO
4	27	M	5	0	1A	0	NO	NO
5	29	F	8	0	2A + 1B	0	NO	NO
6	29	F	5	2	1A + 3A	1A	YES	NO
7	30	F	7	0	1A	0	NO	NO
8	32	F	7	0	1A	0	NO	NO
9	33	M	7	0	1B	0	NO	NO
10	33	M	7	2	1B + 2A	1A	YES	NO
11	33	F	5	2	1B + 3A	1A	YES	NO

## Data Availability

Not applicable.
